# Control of Local Intracellular Calcium Concentration with Dynamic-Clamp Controlled 2-Photon Uncaging

**DOI:** 10.1371/journal.pone.0028685

**Published:** 2011-12-28

**Authors:** Erwin Idoux, Jerome Mertz

**Affiliations:** Biomedical Engineering Department, Boston University, Boston, Massachusetts, United States of America; Dalhousie University, Canada

## Abstract

The variations of the intracellular concentration of calcium ion ([Ca^2+^]_i_) are at the heart of intracellular signaling, and their imaging is therefore of enormous interest. However, passive [Ca^2+^]_i_ imaging provides no control over these variations, meaning that a full exploration of the functional consequences of [Ca^2+^]_i_ changes is difficult to attain. The tools designed so far to modify [Ca^2+^]_i_, even qualitatively, suffer drawbacks that undermine their widespread use. Here, we describe an electro-optical technique to quantitatively set [Ca^2+^]_i_, in real time and with sub-cellular resolution, using two-photon Ca^2+^ uncaging and dynamic-clamp. We experimentally demonstrate, on neurons from acute olfactory bulb slices of Long Evans rats, various capabilities of this technique previously difficult to achieve, such as the independent control of the membrane potential and [Ca^2+^]_i_ variations, the functional knocking-in of user-defined virtual voltage-dependent Ca^2+^ channels, and the standardization of [Ca^2+^]_i_ patterns across different cells. Our goal is to lay the groundwork for this technique and establish it as a new and versatile tool for the study of cell signaling.

## Introduction

Variations in intracellular Ca^2+^ concentration ([Ca^2+^]_i_) play a key role in governing the response of virtually all cell types [1]. The importance of [Ca^2+^]_i_ is particularly marked in neurons where it is instrumental in information processing, plasticity and neurotransmitter exocytosis [2].

While [Ca^2+^]_i_ variations are now routinely imaged by one- or two-photon excited fluorescence [3], [4], few attempts have been made to design tools to actually control these variations. Examples of such attempts have relied on controlling extracellular Ca^2+^ concentration while increasing plasma membrane permeability [5], on using iontophoresis with a sharp electrode [6], or on uncaging chelated Ca^2+^ [7]. Because neuronal physiology is critically sensitive to plasma membrane permeability, the first technique is poorly suited to neurons. The second technique has also been shown to modify neuronal electrophysiology [8] and has the disadvantage that iontophoresis creates a single entry point for Ca^2+^, in contrast to physiological calcium signals that enter via voltage-gated Ca^2+^ channels distributed throughout the membrane. Finally, in the third technique, single-photon ultraviolet pulses trigger the photolysis of a Ca^2+^chelator. Throughout the 90′s, Neher and his group intensively used this to investigate the cellular mechanisms involving Ca^2+^ at the synapse [9]–[11] and in chromaffin cells [12]. However, this method leads to [Ca^2+^]_i_ peaks and decays, which differ from the continuous variations experienced by cells under physiological conditions. Moreover, the excitation volume defined by the UV flash is spatially extended, making it difficult to perform quantitative Ca^2+^ release with high spatial resolution.

The present study describes a new technique that solves many of these problems. Specifically, we have developed a tool to control [Ca^2+^]_i_ with sufficient temporal and spatial resolution to mimic naturally occurring intracellular Ca^2+^ signals. We demonstrate the capabilities of this tool by mimicking voltage-dependent Ca^2+^channels in neurons. The theory of our technique and its experimental design are discussed. We focus on three applications, namely the independent control of the membrane potential and [Ca^2+^]_i_ variations, the functional knocking-in of user-defined virtual voltage-dependent Ca^2+^ channels, and the standardization of [Ca^2+^]_i_ patterns across different cells.

## Results

### Principles of dynamic two-photon uncaging

**Figure 1:**
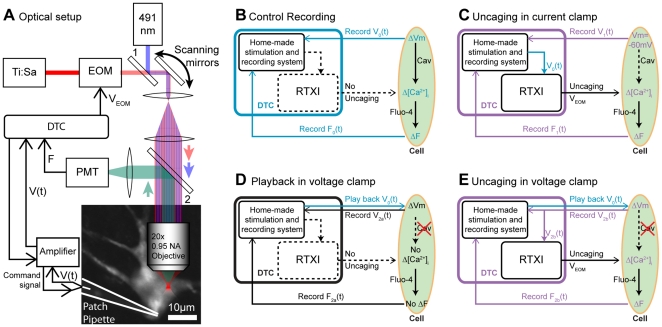
Experimental diagram and design. A. Schematic of the optical setup for DTC. Uncaging was performed by a mode-locked Ti:sapph laser beam (734 nm), the power of which was controlled by an electronic-optic modulator (EOM, Conoptics M350-80). The laser focus was scanned in a closed, curvilinear path along the inner membrane surface of the patched neuron. The patch electrode delivers caged Ca^2+^ (Ca^2+^-laden DM-nitrophen, 1.51 mM) and fluo-4 (77 µM, fluorescent Ca^2+^ sensor). ΔF/F is monitored using a 491 nm DPSS laser (Cobolt Calypso) or a 488 nm argon laser (JDS Uniphase), co-aligned with the uncaging laser through dichroic mirror 1 (see Methods). B. Control case with no uncaging: the reference membrane potential V_0_(t) and fluorescence F_0_(t) are recorded. C. Application 1: V_0_(t), the membrane potential sequence recorded in B. , is used as the input command in RTXI to compute the laser power required for the desired Ca^2+^ uncaging, while the cell is maintained hyperpolarized to prevent endogenous [Ca^2+^]_i_ variations. V_1_(t) and F_1_(t) are recorded by DTC. D. Application 2_a_: To test for residual endogenous Ca^2+^ influx, V_0_(t) is played back into the cell without any uncaging. Since the amplifier is in voltage-clamp mode, V_2a_(t) is recorded and should be identical to V_0_(t). E. Application 2_b_: Similarly, V_0_(t) is played back, but the uncaging is driven by the measured membrane potential of the neuron (V_2b_(t)).

Our approach is two-pronged : we spatially confine the Ca^2+^ release from the chelator DM-nitrophen [13], [14] using two-photon uncaging, and we control its dosage and dynamics in real time by dynamic-clamp [15]. Together, these two techniques provide dynamic two-photon calcium control (DTC) and enable us to liberate ions with similar local concentration and temporal dynamics as would an endogenous voltage-gated Ca^2+^ channels (see Fig. 1a).

Based on the optics in our setup, our two-photon excitation focal volume is approximately 0.5 fL (see Equations 9a and b). This small focal volume can provide targeted Ca^2+^ release, or it can be scanned within the soma, in the vicinity of the cell membrane. This second option was chosen for our purposes since it more closely mimics physiological trans-membrane Ca^2+^ entry and reduces the chance of a non-negligible local depletion of cage complex (cf. Discussion). Within the range of laser intensities used for our two-photon photolysis, the number of released Ca^2+^ ions is proportional to the square of the laser power (cf. Equations 7 and 8 and ref. [16]).

While two-photon Ca^2+^ flash uncaging in neurons has been demonstrated in the past, DTC provides analog uncaging that is tightly regulated. Since the number of Ca^2+^ ions entering through channels is directly proportional to the electrical current flowing through these channels, a dynamic-clamp plug-in determines, in real-time, the laser power required to release this amount of Ca^2+^. We begin by first programming a Hodgkin-Huxley conductance to model the voltage dependent gating properties of a population of user-defined Ca^2+^ channels. The dynamic-clamp then uses it to compute the current at the measured membrane potential. Instead of directly injecting this current into the cell via the patch electrode, as is done in the standard implementation of dynamic-clamp, we calculate the number of Ca^2+^ ions required to bear such a current and adjust our laser power accordingly. In this manner, the influx of Ca^2+^ is the same as that expected from the Ca^2+^ channels. Moreover, by sweeping our two-photon excitation along the inner surface of the cell membrane, we closely mimic the expected spatial distribution of the Ca^2+^ entry.

To verify that DTC can simulate endogenous [Ca^2+^]_i_ variations, we monitored these with fluorescence imaging using fluo-4 as the Ca^2+^ indicator and a second laser (Fig. 1A). The similarity between endogenous and photolytic [Ca^2+^]_i_ was evaluated by first recording spontaneous and/or induced membrane potential variations and their associated fluo-4 fluorescence (subsequently referred to as “F”). We then played back the membrane potential recordings to control Ca^2+^ uncaging. Finally, the cross-correlation between the endogenous and the photolytically-induced variations of ΔF/F was evaluated at zero time lag. A cross-correlation of 100% means both signals have exactly the same dynamics, whereas 0% means they are independent.

### Experimental validation of dynamic two-photon uncaging

To demonstrate the capabilities of DTC, we concentrate here on three applications.

**Figure 2:**
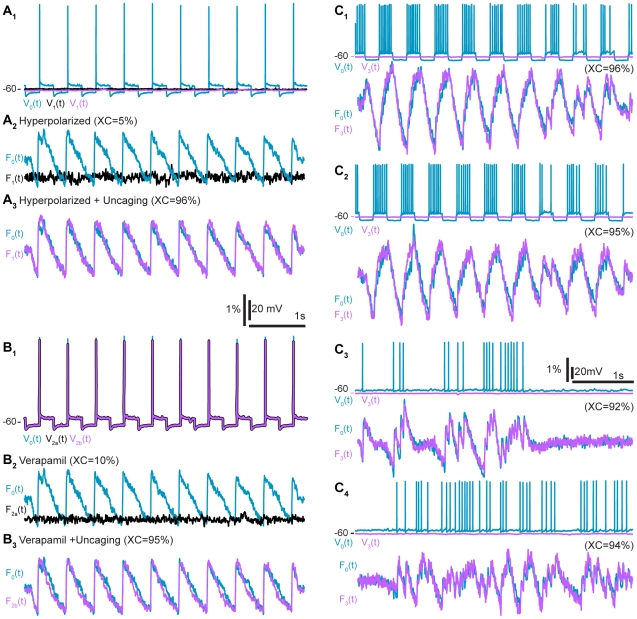
Experimental results. A. Application 1, demonstrating the uncoupling of [Ca^2+^]_i_ and membrane potential variations. Blue, control: F_0_(t)/V_0_(t); black, hyperpolarization without uncaging: F_1_(t)/V_1_(t); magenta, hyperpolarization with uncaging: F_1_(t)/V_1_(t). A_1_ Membrane potential variations. A_2_ Ca^2+^-induced fluorescence variations (F_0_(t) and F_1_(t)) are not significantly correlated. A_3_ Same, but with uncaging. B. Application 2, demonstrating Ca^2+^ channel function restoration. B_1_ Membrane potential variations measured and played back -- the three superimposed traces are identical. B_2_ Fluorescence traces for control (blue F_0_(t)) and without uncaging (black, F_2a_(t)) -- these were not significantly correlated. B_3_ Same, but with uncaging (magenta F_2b_(t)). C Application 3: standardization of [Ca^2+^]_i_ variations across neurons. F_0_(t) and V_0_(t) were recorded in one cell while the uncaging (F_3_(t)) was performed in another that was kept hyperpolarized (V_3_(t)). The same parameters for the conductance model were used for C_1-4_ and endogenous dynamics were reliably reproduced in all cases (see Table 2).

First, we demonstrate that DTC can effectively dissociate [Ca^2+^]_i_ and membrane potential variations. To date, only Ca^2+^channels blockers have been effective at decoupling these by blocking the Ca^2+^ influx altogether for a particular sub-population of channels, effectively preventing the Ca^2+^ influx from depending on membrane potential [17]. We show that DTC enables just the opposite: controlled variations of [Ca^2+^]_i_ while the membrane potential is held constant. To begin, the membrane potential (V_0_(t)) and fluorescence variations (F_0_(t)) are recorded when the neuron is spiking (Fig. 1B and 2A_2_). These recordings serve as a reference. The neuron is then hyperpolarized below the activation threshold of L-type Ca^2+^ channels, causing these endogenous channels to close and the Ca^2+^-associated fluorescence signal to flatline (2A_2_, F_1_). As expected, F_0_ and F_1_ are not significantly correlated and XC_0,1_(0) is down to 8%±15% (n = 56). When DTC is activated (Fig. 1C and 2A_3_), the uncaging restores the [Ca^2+^]_i_ variations with dynamics significantly correlated to the endogenous ones: XC_0,1_(0) = 88%±8% (n = 56). The cross-correlations with and without uncaging are also verified to be significantly different from each other (p<0.001). Thus, DTC effectively simulates the endogenous L-type Ca^2+^ channels even though the membrane potential is held below threshold.

In a second application, the endogenous Ca^2+^ channels are blocked pharmacologically and DTC is used to functionally reinstate the [Ca^2+^]_i_ variations. To do this, we first record a reference, as before (Fig. 1B). Then, 40 µM of verapamil or nifedipine are added to the bath for 15 minutes, thus blocking L-type Ca^2+^ channels [18]. The reference membrane potential variations are then played back as the voltage command in voltage clamp (Fig. 1D), while the resultant fluorescence is monitored. Without DTC, the endogenous fluorescence variations are greatly reduced or even completely canceled, as expected (Fig. 2B_2_, XC_0,2a_(0) = 32%±20%, n = 17). With DTC, the [Ca^2+^]_i_ variations are restored to their original level (Fig. 1E and 2B_3_ XC_0,2b_(0) = 86%±13%, n = 17, significantly higher than playback without uncaging, p<0.001). That is, DTC effectively re-introduces “virtual” L-type Ca^2+^ channels in the soma to replace those that were pharmacologically blocked. Interestingly, uncaging while the Ca^2+^ channels are blocked leads to similar results as uncaging under hyperpolarization but without blockers (i.e. experiment 1), as shown by the high cross-correlation for this subset (XC_0,1_(0) = 91%±4%, n = 17, p = 0.243).

In a third application, DTC is used to standardize the [Ca^2+^]_i_ variations between different cells. The procedure is the same as for application 1 (Fig. 1C), except that the membrane potential variations recorded as a reference in one neuron (neuron_0_) are played back in a different neuron (neuron_1_) that is hyperpolarized. Because the cytoplasms of both neurons are dialyzed by the content of the patch pipette, the Ca^2+^ buffers inside both neurons are also quantitatively and qualitatively similar: the playback of the membrane potential of neuron_0_ thus induces the same [Ca^2+^]_i_ variations in both neuron_0_ and neuron_1_ (Fig. 2C_1_ XC_0,3_(0) = 93%±6%, n = 108).

Finally, to verify the robustness of the L-type Ca^2+^ conductance model and its ability to reproduce arbitrary [Ca^2+^]_i_ variations, the same model parameters were used to reproduce up to four different sequences in a given neuron. The measured [Ca^2+^]_i_ variations all reached similar levels (e.g. Fig. 2c_1_ to 2c_4_ XC_0,3_(0) = 91%±5% n = 26, 89%±6% n = 26, 87%±9% n = 13 and 91%±3% n = 2, Friedman test, p = 0.194). Reproducibility was further confirmed with back to back sequences, differences being on average 0%±2%, n = 401, p = 0.491 (see Methods).

## Discussion

In summary, DTC provides a dynamic control of [Ca^2+^]_i_ with high enough spatio-temporal resolution to simulate the cellular variations of [Ca^2+^]_i_ caused by endogenous channels. It relies on the regulated release of Ca^2+^ by uncaging with two-photon laser, whose power is modulated by a dynamic-clamp plug-in. To our knowledge, such precise control of [Ca^2+^]_i_ dynamics has never been achieved before. DTC therefore provides a new and versatile tool for the study of cellular function, with capabilities that include the dissociation of membrane potential from [Ca^2+^]_i_ variations and the standardization of [Ca^2+^]_i_ patterns through the virtual knock-in of calcium-specific, user-defined conductances.

### Advantages of the technique

DTC is based on two-photon uncaging, which has several advantages: two-photon uncaging both defines a 3D spatially confined Ca^2+^ release volume [19] as well as increases penetration depth in tissue and minimizes photodamage inflicted on non-target cells. Moreover, it offers a wealth of possibilities: in application 2 presented above, DTC not only functionally reinstates pharmacologically blocked channels but also provides full control over both the temporal dynamics of the [Ca^2+^]_i_ variations, and their spatial location. Whereas blockers act indiscriminately in both the soma and dendrites, DTC can be applied more selectively by targeting the laser focus to either or both, enabling the user to gauge the effect of subcellular location of Ca^2+^ influx. Thus, DTC provides the tool to test hypotheses related to key properties of Ca^2+^ signaling, such as the functional importance of subcellular domains, or the impact of slowing or speeding the rate of Ca^2+^ influx.

### Choice of the Ca^2+^ cage and of the Ca^2+^ fluorescent dye

While several cages have been reported in the literature [14], only two of them are currently commercially available: DM-nitrophen and NP-EGTA. Because DM-nitrophen is EDTA based while NP-EGTA is EGTA based, their chelating properties are slightly different. The EGTA core of NP-EGTA is highly selective at binding Ca^2+^ over Mg^2+^ (Kd_Ca_ 80 nM vs. Kd_Mg_ 9 mM), unlike DM-nitrophen (5 nM vs. 25 µM). Therefore, with physiological concentrations of Mg^2+^ in the pipette (∼0.5 to 1 mM [20]), Ca^2+^-laden DM-nitrophen exchanges Ca^2+^ for Mg^2+^, leading to an increase in the resting [Ca^2+^]_i_ from 100 nM to 5 µM and a mixture of Ca^2+^ and Mg^2+^ uncaging [21]. These problems can be avoided with the use of NP-EGTA or, as was proposed before [22] and done here, by removing Mg^2+^ from the internal pipette solution. Then, DM-nitrophen offers numerous advantages over NP-EGTA: it has a higher affinity for Ca^2+^ when intact (5 vs. 80 nM) and a lower affinity when photolyzed (3 vs. 1 mM), and it exhibits a higher two-photon cross-section (0.01 vs. 0.001 GM). The lower affinity allows DM-nitrophen to be loaded above 95% for [Ca^2+^]_i_ ≈100 nM, while under the same conditions (same [Ca^2+^]_i_ and same amount of free Ca^2+^ buffer), NP-EGTA could only be loaded slightly above 50% . Moreover, NP-EGTA would be restricted to very low concentration (around 200 µM) to allow signal monitoring with 100 µM fluo-4. The combined effect of low concentration of caged NP-EGTA and lower two-photon cross-section means that NP-EGTA would require more than 10 times more optical power (536 mW instead of 47 mW to attain 1000 pA Ca^2+^ current), which would be difficult to achieve with our current setup, in addition to being potentially detrimental to the preparation. A possible solution is to increase both NP-EGTA and fluo-4 concentrations, though this may be ill-advised for two reasons. First, the NP-EGTA would still be only half loaded, which may cause the Ca^2+^ to be released only transiently rather than continuously, as freshly released Ca^2+^ is likely to be quickly recaptured by non-photolyzed neighboring empty cages [23]. An increase in NP-EGTA and fluo-4 concentrations would also increase the concentration of free Ca^2+^ buffer, which might distort the endogenous Ca^2+^ signal [24], [25]. For example, to lower the laser power from 536 to 107 mW would entail about a 3.3 mM increase in free Ca^2+^ buffers (free NP-EGTA + free fluo-4). Thus, we conclude that whenever Mg^2+^ is not crucial for the study in question (the case here), then DM-nitrophen seems to be a better choice than NP-EGTA for the application of DTC.

Local depletion is yet another risk to be aware of when uncaging Ca^2+^. As noted above, only 47 mW of laser power is required to attain a 1000 pA Ca^2+^-current when using DM-nitrophen, while a typical current in mitral cells is usually less than 150 pA [18]. To reproduce such a typical current between sequential dynamic-clamp updates would locally consume about 10% of the caged Ca^2+^ pool in a static release volume, i.e. if the laser were kept parked at one spot. Because the laser is instead scanned along the inner membrane surface, the swept release volume is about 90 times larger than the focal volume (about 40 fL for a 15 µm diameter cell). Therefore, the local depletion is about 0.1% and can be readily replenished by diffusion from non-illuminated neighboring regions, as experimentally verified when comparing back to back sequences.

As noted above, our Ca^2+^ reporter is fluo-4. This was chosen because it provides several advantages compared to other available Ca^2+^ reporters. First, with its absorption peak at 494 nm [26], it is almost optimally excited by our 491 nm CW laser, while not excited by the two-photon laser (734 nm). It is furthermore compatible with DM-nitrophen since the excitation wavelength of nm also prevents unwanted uncaging since it does not photolyze DM-nitrophen [13]. Second, its K_d_ (345 nM) makes fluo-4 one of the best choices to monitor both single elementary events (such as the [Ca^2+^]_i_ increase after an action potential) and larger trends in Ca^2+^ variations [26], [27]. Third, because we use a cell-impermeant version of fluo-4, it is likely to be homogeneously distributed throughout the cytoplasm, with little inhomogeneity due to compartmentalization [27].

### Limitations of the technique

Since DTC uncaging is light-based, its spatial resolution cannot be better than the diffraction limit. The water-immersion objective used here has a numerical aperture of 0.95, which is close to the maximum commercially available while remaining compatible with patch clamp (i.e. with a working distance over 2 mm). With our setup (see Table 1), the release volume is a prolate spheroid with equatorial and axial radii of 0.4 and 1.4 µm respectively. The size of our focal volume is thus on the order of the femtoliter. This is clearly much larger than the volume of a Ca^2+^ spark or sparklet from a single channel [28], [29], meaning that DTC cannot replicate the spatial confinement of a single channel Ca^2+^ influx. Nevertheless, when considering the Ca^2+^ influx produced by a local population of channels (the channels distributed in the plasma membrane of the soma in our case), their averaged signals over the time scale of interest can be faithfully reproduced, as experimentally demonstrated here. Working with a population of channels allows us to use the deterministic Hodgkin-Huxley formalism to model this global conductance. If needed, a random component can also be introduced to mimic population stochastic response [30].

**Table 1:** Complete set of *parameters for power modulation*.

Parameter	Value	Meaning
λ	734 nm	Laser center wavelength
f	80 MHz	Repetition rate of the laser
t_p_	100 fs	Laser pulse width
g_p_^(2)^	0.59	Gain factor for the Sech^2^ pulse [34]
σ_2_	0.013 GM	2-photon cross-section[16]
n	1.33	Refractive index of water
NA	0.95	Olympus 20× XLUMPLANFI water immersion objective
[CCa^2+^]_0_	1.513 mM	Concentration of caged calcium. See Methods

How fast can our DTC be pushed? In this set of experiments, we use a refresh rate of 10 kHz for the dynamic-clamp. This is about one order of magnitude faster than the fastest time constant of the conductance we mimic (cf. Table 2) and is thus sufficient for our applications. Faster refresh rates could easily be achieved with our computer, however one must bear in mind that the photolysis mechanism itself cannot be sped up: the maximum release rate for DM-nitrophen is about 40 kHz, whereas it reaches about 70 kHz for NP-EGTA [14].

**Table 2:** Conductance parameters.

Gate	r (1/mV)	s (1/mV)	t (ms)	v (mV)
a (activation)	−0.04	0.025	3	3
i (inactivation)	−0.02	−0.015	120	−30

Finaly, we emphasize here that DTC should not be regarded as a “calcium-clamp” for two reasons. First, a proper clamp would involve a feedback mechanism to maintain [Ca^2+^]_i_ at a set value. To mimic voltage-dependent Ca^2+^ channels, DTC does not rely on feedback, and instead is based only on a feed-forward mechanism, as illustrated in figure 1C. That is, the actual value of [Ca^2+^]_i_ is never used to compute how much Ca^2+^ is to be released. Second, a proper clamp would involve a way to actively modulate the Ca^2+^ concentration both upward and downward. In our case, DTC only achieves the upward modulation: the downward modulation is ensured by the cell Ca^2+^ clearance mechanisms, as is the case when Ca^2+^ enters the cell through voltage dependent Ca^2+^ channels. While in principle it may be possible to introduce an active Ca^2+^ scavenger in addition to our Ca^2+^ chelator, the only photoactivatable scavenger commercially available is, to our knowledge, Diazo-2 (available from Invitrogen [31]), whose activation spectrum overlaps that of both DM-nitrophen and NP-EGTA, thus precluding an independent control. For these reasons, DTC cannot be regarded as a bona-fide “calcium-clamp”.

### Extension of the tool to other Ca^2+^ sources and to more caged compounds

It should be noted that DTC can, in principle, be applied to quantitatively control the release of any caged compound with adequate two-photon cross-section. For example, another mechanism through which Ca^2+^ enters the cytoplasm is CICR (Ca^2+^-induced Ca^2+^ release) and DTC can be programmed to mimic this as well, provided an appropriate CICR model is implemented. In this case, the RTXI input would not be the membrane potential but rather a measure of the actual [Ca^2+^]_i_ , for example obtained by fluorescence monitoring with a Ca^2+^-sensor. With the appropriate Ca^2+^ cage, such an implementation of DTC would be all-optical and allow simultaneous multicellular control. Finally, DTC need not be restricted to caged Ca^2+^. For example, caged neurotransmitters can be used to reproduce localized synaptic release with controlled dynamics. Alternatively, caged-second messengers such as inositol trisphosphate can be used to study second messenger dynamics in sub-cellular compartments.

In short, DTC is a general technique with many potential ramifications. Our goal in this study is to lay the groundwork for this technique, with the hope of establishing it as a new tool for the study of cellular dynamics. Specifically, we have confined our proof of concept to the modulation of Ca^2+^ in neurons, where we show that the combination of a dynamic-clamp with two-photon uncaging enables the release of caged compounds with an unprecedented degree of control and spatiotemporal resolution. This technique is robust and easy to implement with standard two-photon microscopes, making it attractive as a general tool for the study chemical signaling in cells.

## Materials and Methods

### Electrophysiology

The following protocol was approved by the Boston University Institutional Animal Care and Use Committee (IACUC Protocols # 07–014 and 10–021) and everything was done to minimize the number of animals and their suffering. We prepared parasagittal olfactory bulb slices (350 µm) from weaned sub-adult *Long-Evans* rats (age: P21 to P41, median P32). Briefly, after a deep, isofluorane-induced anesthesia, confirmed by absence of reaction to toe pinching, the animal was decapitated and the head submerged in ice-cold sucrose aCSF (see composition below). The olfactory bulbs were removed, sliced with a Vibratome 1500 (formerly sold by Vibratome, now Leica), and placed in regular aCSF bubbled with 95% O_2_-5% CO_2_ to attain a pH of 7.3–7.4. Slices were kept at room temperature until individually transferred into the recording chamber where experiments were carried out. One neuron was patched per slice, with a borosilicate glass pipette pulled on a Sutter P97 puller from BF120-69-10 capillaries (3–6 MΩ).

### Solutions

Both the room temperature incubation and subsequent recording at 31°C used artificial cerebro-spinal fluid (aCSF) composed of (in mM): 120 NaCl, 25 NaHCO_3_, 1 NaH_2_PO_4_, 2.5 KCl, 10 glucose, 2 MgCl_2_, 1 CaCl_2_. The sucrose aCSF used for the dissection and slicing is the same, but with the 120 mM NaCl replaced by 240 mM sucrose and the MgCl_2_ increased to 3 mM to compensate for the removal of CaCl_2_. Both solutions have the same osmolality (∼295 mosm/kg, Wescor Vapro 5520) and pH (7.3–7.4). L-type calcium channels [18] were blocked by addition in the aCSF of 40 µM of verapamil or nifedipine while the cell was depolarized so that it spiked for 15 minutes. The solution used to fill the pipette was composed of (in mM) 135 K-gluconate, 5 KCl, 7.5 HEPES, 2.5 NaHEPES, 4 K_2_ATP, 0.47 Na_x_GTP, 1.56 CaCl_2_, 1.585 DM-nitrophen and 0.1 K_5_fluo-4. Therefore, the concentrations of loaded DM-nitrophen, free Ca^2+^, and free fluo-4 are, respectively 1.51 mM, 104 nM and 77 µM.

All chemicals were obtained from Sigma-Aldrich, with the exception of DM-nitrophen and fluo-4, obtained from Invitrogen.

### Stimulation methods: playback

To ensure that the Ca^2+^ fluorescence traces could be compared between runs and conditions, the membrane potential variations had to be kept identical between different traces. Therefore, our custom-made stimulation and recording program (Matlab and Labview) included a playback mode. This mode outputs previously-recorded sequences of membrane potential variations through a National Instruments card (PCI-6221 with a BNC-2110) and sends them to either the amplifier or dynamic-clamp setup (see Fig. 1).

### Microscope

Our upright two-photon microscope was custom-built and described elsewhere [32]. The rest of the setup is described in Fig. 1A. The ΔF/F was calculated from the fluorescence signal (F) recorded by a PMT (Hamamatsu H7422PA-40).

### Analysis: cross-correlation

To compare the dynamics of two fluorescent traces of N points (F0 and F1), we evaluated their cross-correlation [33] at zero lag, XC_0,1_ (τ = 0). If µ0 and σ0 are respectively the mean and standard deviation of F0 (similarly for F1), then XC_0,1_ (τ) is given by



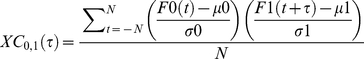



Furthermore, two traces are significantly correlated with a 5% threshold [33], only if



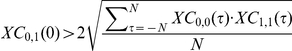



Unless stated otherwise, we used only significantly correlated traces. To test for reproducibility between sequences, some sequences (n = 401) were repeated back to back, separated by a 1s interval. Since the differences were not statistically significant (p = 0.49), only the highest of the two cross-correlation coefficients was kept for subsequent analysis.

### Dynamic-clamp and model for uncaging dynamics

Experiments were performed using the Real-Time eXperiment Interface (RTXI; www.rtxi.org), connected through a National Instruments card PCI-6040E and a plug-in with a model of the L-type Ca^2+^ conductance. This samples the membrane potential V_(t)_ and calculates the value of the conductance every 100 µs (10 kHz refresh rate). The corresponding current, namely I_Ca_(V_(t)_), is then computed based on V_(t)_ and a locally applied Ohm's law. Since an ionic current corresponds to a number of charges flowing through a surface per unit of time, we thus determine the number of calcium ions (ΔN_Ca_) required to produce such a current, for a given time step.

The time step (Δt_DC_ = 100 µs) is set to be the sampling period of the voltage by the dynamic-clamp software, which is much faster than typical time constants involved in the dynamics of the Ca^2+^conductances (see Table 2). The surface in question is that of the plasma membrane of the soma, however it could be chosen elsewhere, depending on the experiment (e.g. it could be set to correspond to a portion of dendrite, subcellular compartment, etc.).

The above provides us with the first relationship between the current and the number of Ca^2+^ ions:



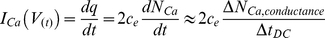
(1)


where c_e_ is the elementary charge and V_(t)_ the membrane potential.

The laser in our system is operated at an intensity I_L_ far below the onset of pulse saturation [16], [19]. The rate of release of Ca^2+^ions (R_Ca_(I_L_,x,y,z)) is therefore linearly proportional to the actual caged Ca^2+^available ([CCa^2+^]_0_), at any point in space (x,y,z) [16]. That is,



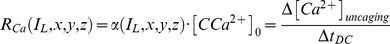
(2)


where α, the photolysis rate, is defined as



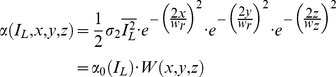
(3)


where σ_2_ is the two-photon photolysis cross-section and we have adopted a 3D Gaussian model for the focal volume, with lateral and axial waists *w_r_* and *w_z_* respectively. We further adopt the notation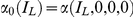. In writing Eq. 2, we have made the assumption that [CCa^2+^]_0_ is roughly a constant, i.e. not depleted by the uncaging process itself. This assumption is justified in the discussion and experimentally confirmed.

The principle of DTC is to match ΔN_Ca,uncaging_ and ΔN_Ca,conductance_ during the interval Δt_DC_. The total number of Ca^2+^ ions (ΔN_Ca_) liberated in the release volume (V_r_) can be expressed as



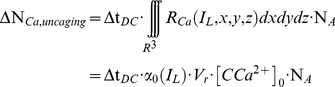
(4a)




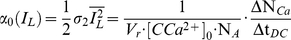
(4b)




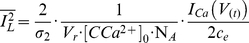
(4c)




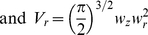
(5)


where N_A,_ Avogadro's number, converts the result of the volume integral from moles into number of ions.

Since 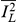 is directly proportional to I_Ca_, the current calculated by the dynamic-clamp, we then control the intensity of the laser beam to reproduce the Ca^2+^ variations in the cell in real time. The mean of the squared intensity 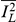 is linked to the square of the mean intensity (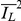), through the “two-photon advantage factor” resulting from the pulsed nature of the laser beam used in the experiments [19], [34]:



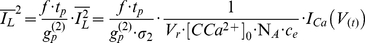
(6)


where *f* is the laser repetition rate, *t_p_* is the pulse duration and g_p_^(2)^ is the second-order temporal coherence factor.

An electro-optic modulator (EOM) controls the laser power, which, for wavelength λ, is given by



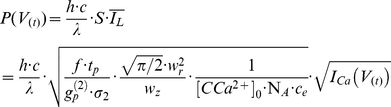
(7)


since the area of the beam at the focal plane is 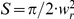. From Equation 7, we observe that the only time varying parameter is I_Ca_(V_(t)_) (see conductance model below), the rest being just constant scaling factors.

Finally, the input voltage range of the EOM (0 to V_max_) is adjusted to provide a linear control of the optical power (0 to P_max_). The voltage output of RTXI then becomes



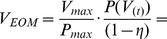
(8)


where η is he power loss throughout the system, which was evaluated to be 75% for our setup. Specifically, the 326 mW power exiting the laser was reduced to 82 mW at the focal plane because of losses mostly due to the EOM and the dichroic mirrors in our setup (cf. Fig.1 1: DMLP567 from ThorLabs, 2: Custom-made by Chroma 540DCRB, reflectance band: 540±40, transmittance 94–95% at 488/491 nm and 88–92% in the 734±4 nm band).

*w_r_* and *w_z_*, the radial and axial waists of the release volume, were calculated using [19]:



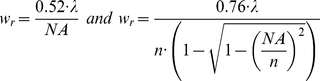
(9ab)


with NA being the objective numerical aperture and *n* the index of refraction of water.

See Table 1 for parameter values.

### Conductance model

To program the plug-in that models the L-type calcium conductance, we adopted an approach similar to one used previously [35], using standard Hodgkin-Huxley equations [36], with parameters adapted from Bhalla and Bower [37]. The equations are summarized as



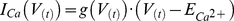
(10)


where E_Ca_^2+^ is the reversal potential of Ca^2+^ and g the conductance, expressed as



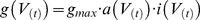
(11)


with a(V(t)) being the activation gate, i(V(t)) the inactivation gate, and g_max_ the maximal value of the conductance. The gates *a* and *i* are modeled as typical Hodgkin-Huxley gates:



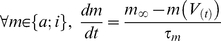
(12)




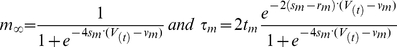
(13ab)


See Table 2 for parameters.

### Statistical analysis

To determine whether the differences observed in XC_0,1_(0) between conditions were statistically significant, we used the Wilcoxon signed rank test, unless stated otherwise. This non-parametric test was chosen since some sets were not normally distributed (as tested with a Shapiro-Wilk test). However, the medians and means were close enough to report the results as mean ± S.D as usual.
